# Inertial Sensor-Based Methods in Walking Speed Estimation: A Systematic Review

**DOI:** 10.3390/s120506102

**Published:** 2012-05-10

**Authors:** Shuozhi Yang, Qingguo Li

**Affiliations:** Department of Mechanical and Materials Engineering, Queen's University, Kingston, ON K7L 3N6, Canada; E-Mail: yangs@me.queensu.ca

**Keywords:** walking speed, ambulatory, spatio-temporal parameters, biomechanics, inertial sensors, gait segmentation, review

## Abstract

Self-selected walking speed is an important measure of ambulation ability used in various clinical gait experiments. Inertial sensors, *i.e.*, accelerometers and gyroscopes, have been gradually introduced to estimate walking speed. This research area has attracted a lot of attention for the past two decades, and the trend is continuing due to the improvement of performance and decrease in cost of the miniature inertial sensors. With the intention of understanding the state of the art of current development in this area, a systematic review on the exiting methods was done in the following electronic engines/databases: PubMed, ISI Web of Knowledge, SportDiscus and IEEE Xplore. Sixteen journal articles and papers in proceedings focusing on inertial sensor based walking speed estimation were fully reviewed. The existing methods were categorized by sensor specification, sensor attachment location, experimental design, and walking speed estimation algorithm.

## 1. Introduction

Walking is one of the most important daily activities for people to get from one place to another. Not surprisingly, spatial and temporal gait parameters during walking (*i.e.*, walking speed, stride length, stride frequency, and gait asymmetry) have been extensively studied for healthy and pathological populations. Besides these parameters, self-selected walking speed has long been recognized as a proxy measure of ambulation quality and is used to quantify the progress of gait rehabilitation [1–4]. Traditionally, a stopwatch has been used to determine the average speed during walking through a known distance, which is commonly practiced in clinical settings. To obtain more accurate results and instantaneous walking speed, laboratory-based camera based motion tracking systems (*i.e.*, OptoTrack, NDI) and instrumented walkways (*i.e.*, the GAITRite system) have been introduced to determine the individual stride length and stride frequency as well as the walking speed [5]. Despite their accuracy, these systems are restricted in laboratory settings due to their size and cost.

With the advancement of microelectromechanical systems (MEMS), inertial sensors (accelerometers and/or gyroscopes) became more and more popular in physical activity monitoring because of their portability, improved performance, and low cost [6]. Since the 1990s, many inertial sensor-based methods have been developed for estimating spatio-temporal parameters during walking. The spatio-temporal parameters reported in these studies include walking speed, stride length, and total distance walked. The estimation of the last two parameters is equivalent to estimating walking speed, but with a different emphasis. The stride length defines the travel distance in one stride, and the walking speed can be calculated by multiplying the associated stride frequency. The stride length tends to be used for evaluating gait variability between walking trials. On the other hand, the total distance walked quantifies the distance covered, and the average walking speed can be easily calculated by dividing the distance traveled with time spent. Although the selection of parameters are different between studies, the essence is the same in estimating the distance covered in a fixed time interval. Therefore, this review does not distinguish stride length estimation and walking distance estimation from walking speed estimation.

**Figure 1.**
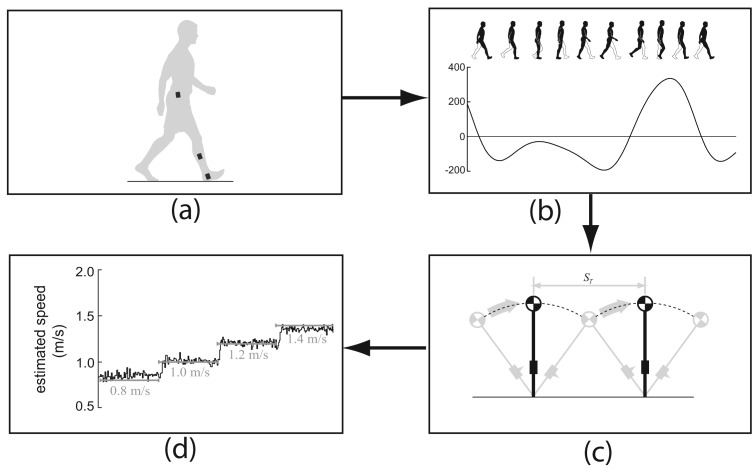
Generic walking speed estimation method. (**a**). Inertial sensors (accelerometer and/or gyroscope) are attached to different parts of the user. (**b**). Inertial sensors measure the accelerations and/or angular velocities which contain information related to the walking speed. (**c**). A walking speed estimation algorithm extracts the walking speed information from these sensor measurements. (**d**). Different walking speed are distinguished as outputs of the algorithm.

As the name suggests, an accelerometer is a device for measuring accelerations, including those induced by gravity. A gyroscope measures angular velocity. The combinations of these sensors are referred to as inertial measurement units (IMU). During walking, body segments undergo a cyclic motion and the movement pattern of each segment repeats every stride cycle. The cyclic motion of a body segment induces periodic acceleration and angular velocity changes, which can be sensed by the attached inertial sensors. The embedded features in the measurements enable the possibility in estimating walking speed. A generic structure of a walking speed estimation method is illustrated in Figure 1. Inertial sensors can be attached to different parts of the body (Figure 1(a)) and measure different signals (Figure 1(b)). With these measurements, different models and methods can be used to estimate walking speed (Figure 1(c)). Under this generic structure, a wide range of inertial sensors and configurations have been adopted in estimating walking speed, and it is certainly of interest to examine how they were utilized and how well they performed. There are comprehensive reviews in the area of wearable sensors for gait analysis [7], reviews on inertial sensors in monitoring lower limb biomechanics [8], and in the gait event detection [9]. However, there is no review on a current status of inertial sensors in walking speed estimation, which is the primary purpose of this paper.

## 2. Methods

### 2.1. Review Questions

We systematically reviewed the literature regarding the inertial sensor-based walking speed estimation methods, and attempted to answer the following questions: (1) What are the existing inertial sensor based walking speed estimation methods (including stride length estimation and walking distance estimation)? (2) What types of inertial sensors were used in related studies? (3) Where were the inertial sensors attached? (4) How were the experiments conducted? and (5) How was the performance of these studies?

In order to answer all these questions, we reviewed the literature on inertial sensor based walking speed, stride length and walking distance estimation methods.

### 2.2. Article Selection

**Figure 2.**
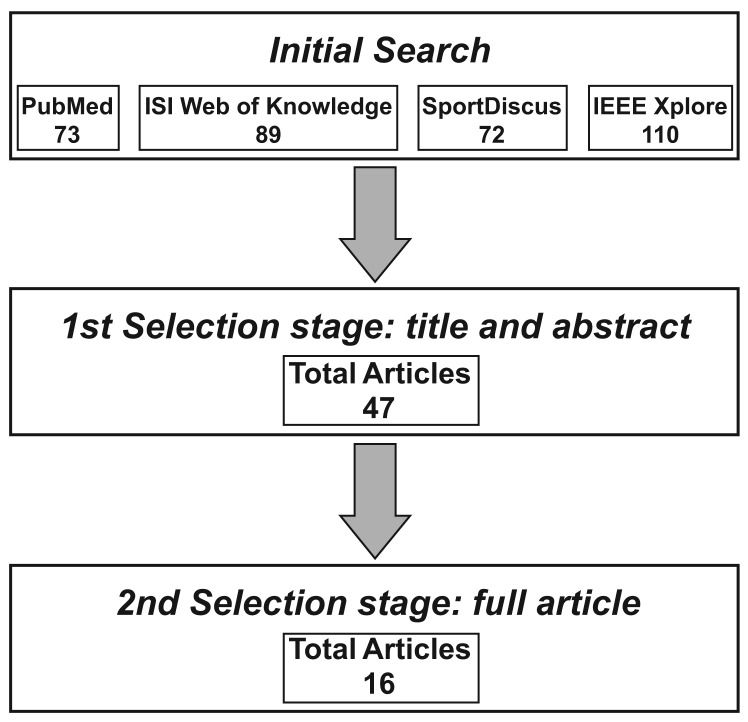
Article review procedures. After the initial search, the title and abstract were reviewed first to exclude unrelated articles. The full articles were then retrieved and reviewed with the detailed inclusion/exclusion criteria. 16 articles were finally included in this review.

The research method is graphically depicted in Figure 2 for better understanding of the procedure. We systematically searched for published journal articles and papers in proceedings in PubMed (from 1950), ISI Web of Knowledge (Science Citation Index Expanded, from 1899; Social Sciences Citation Index, from 1956; Art & Humanities Citation Index, from 1975), SportDiscus (from 1950) and IEEE Xplore (from 1950) at the first week of July in the year 2011. These four electronic engines/databases were chosen because of their popularity and their coverage of literature in engineering, medicine and biomechanics. The searched keyword string was “*(assessment OR estimation OR calculation OR computation OR measurement) AND (inertial sensor OR accelerometer OR gyroscope OR inertial measurement unit) AND (speed OR velocity OR step length OR stride velocity OR stride length) AND (walking)*” for appearance in the title, abstract and keyword fields of the articles. The initial total number of identified articles was 344. The title and the abstract of each article was read carefully for the first selection stage, and unrelated and duplicated articles were excluded, which reduced the number of articles to 47. In the second selection stage, these 47 full articles were retrieved from the Queen's University library system and completely reviewed. A total of 16 full articles were ultimately included in this review. The inclusion criteria were as follows: (1) the study involved inertial sensors, and (2) the study reported walking speed, stride length or walking distance estimation results. However, as this review focuses on the method development, articles only containing the following contents were excluded: (1) performance evaluation of a commercial available product without revealing the walking speed estimation method, (2) performance comparison between existing methods, and (3) applications based on previous reported methods.

## 3. Results

### 3.1. Sensor Specification

Large varieties of inertial sensors are currently available on the market, ranging from uniaxial accelerometers/gyroscopes to IMUs with 6 degrees of freedom (6DOF). The measurement range of the inertial sensors varied with the specifications, from ±2 g to ±50 g for accelerometers and from ±150°*/s* to ±1,000°*/s* for gyroscopes. The inertial sensor measurements were sampled and filtered with different frequencies. Depending on the purpose of the study and the system design, different inertial sensors and sensor configurations were adopted. Table 1 in Appendix shows the detailed specifications of sensors used in these studies. No clear rationale in sensor selection has been reported in the reviewed articles. As a general rule, the section of sensor type (accelerometer and/or gyroscope) is dependent on the walking speed estimation algorithm. For example, the direct integration based approach requires measurements of both acceleration and angular velocity. On the other hand, the selection of sensor measurement range is determined by the location of the sensor, and in most cases, a more distally attached sensor should have a bigger range of measurement.

### 3.2. Sensor Attachment Location

Most body motion during human gait occurs in the lower limbs; therefore, most of the reviewed studies chose to attach the inertial sensors on the thigh and the shank or the foot of the subjects. One study [10] attempted to capture the motion with an accelerometer attached to the chest. Four studies [11–13] utilized sensors attached at the lower spine to estimate the walking speed. One study additionally used a force/moment sensor [14] as an aiding component in the system. Table 2 in Appendix shows the sensor types and the attachment locations used in the reviewed articles. We followed a similar illustration method as proposed in [9]. The effect of sensor attachment location on the quality of the measurements was not discussed in the reviewed studies. The following factors need to be considered when selecting a sensor location. (1) The linkage between the sensor measurement and the walking speed. Even walking at the same speed, the characteristics of acceleration and angular velocity are different from location to location. A walking speed estimation algorithm extracts walking speed information from these characteristics and therefore is sensor location dependent. The feasibility of deriving walking speed information from sensor measurements needs to be considered first in selecting a specific sensor location. (2) The relative movement between the sensor and the body segment. As the sensors are typically attached to the body segment with a strap or tape, relative motion is unavoidable and will cause discrepancy between the measured acceleration and angular velocity and those of the body segment. The differences ultimately affect the walking speed estimation accuracy and robustness of the algorithm. (3) Robustness to the disturbance caused by the abnormal motion. In each walking speed estimation method, certain assumptions have been made under which the algorithm will function properly. Any abnormal motion that deviates from the assumption will generate errors in the estimated walking speed. As an example, a sensor attached to the foot may be affected by the abnormal foot motion, such as the equinus gait often observed in children with cerebral palsy [15]. A concurrent comparison study demonstrated that the foot attached sensor is prone to walking speed estimation error during toe-out walking as compared with the shank-mounted sensor when using a 2D inertial sensor [16]. The robustness needs to be considered in selecting a sensor location, especially when applying the walking speed estimation method to a population with gait abnormalities.

### 3.3. Experimental Design

Most of the reviewed studies focused on experimental validation with healthy subjects. Some studies aiming to apply their methods in age-related or pathological gait chose to include elderly subjects [17,18], spinal cord injured subjects [19] or patients with prostheses or hemiplegic gait [20]. In some studies, the proposed methods were validated with only one subject [19,21–24]. Although single subject verification might not be sufficient to demonstrate the robustness of the proposed methods, the idea of the proposed methods was clearly explained. Two major forms of experimentation were treadmill walking and overground walking at either preset speed or preferred speed. For those studies that involved elderly or impaired subjects [17–20], the experiments were designed with care while providing reasonable comparison with young/healthy subjects. When compared with healthy young subjects, the walking speed estimation method produced less accurate results for subjects with pathological gait in most cases. Four out of 16 studies [14,18,22,23] concentrated on inertial sensor based personal navigation systems that were capable of monitoring the subjects walking in 3D environments; 12 out of 16 studies focused on the motion of the subjects in the sagittal plane only. The detailed experimental design information is shown in Table 3 in Appendix.

### 3.4. Walking Speed Estimation Algorithm

The algorithms of using inertial sensors to estimate walking speed can be grouped into three categories: (1) abstraction model (three studies: [10,11,13]), (2) human gait model (five studies: [12,17,19,20,25]) and (3) direct integration (eight studies: [14,18,21–24,26,27]).

#### 3.4.1. Abstraction Model

Instead of calculating walking speed based on a certain physical model, some studies decided not to look into the details of the human gait biomechanics, but to abstract the system and construct a black-box model for the complex relationship between the sensor measurements and the walking speed from an information processing point of view.

In 1995, Aminian *et al.* [11] proposed a walking speed estimation algorithm with the aid of artificial neural networks (ANNs) with four acceleration measurements as inputs. In this study, the system consisted of two two-layer ANNs, in which the input acceleration signals were collected from the back trunk (triaxial) and the heel (uniaxial), and the first ANN generated the incline estimates while the second ANN estimated the walking speed. Before the classification phase (walking speed estimation), the training phase (learning process) of each ANN used a set of 360 acceleration signal patterns obtained from treadmill walking experiments, and associated the acceleration signals with the actual walking speed by adjusting the weights and biases of the ANN to minimize the sum squared errors. At the end of the training phase, a black-box ANN model with fixed weights and biases was generated and later used in the classification phase to map the acceleration signals to the walking speed, which achieved a maximum relative error of 16% from the overground walking experiments. A similar method was adopted by Song *et al.* [10]. Song employed a two-stage structure consisting of three ANNs to process the acceleration signals collected from an accelerometer (triaxial) attached to the chest. At the first stage, the walking/running classification network classified the type of gait, either walking or running; at the second stage, the walking neural network (NNW) or the running neural network (NNR) processed the acceleration signals accordingly to estimate walking or running speed. The overall root mean squared error (RMSE) was 0.54 *km/h* based on walking/running experiments at various speed between 4.7 *km/h* and 17.14 *km/h*. Different from the ANNs used in these two studies, Yeoh *et al.* [13] defined the average net acceleration (ANA) of the left and right thighs and estimated the walking speed using a third-order polynomial model. Before the walking experiment, a sufficient amount of acceleration signal data at various walking speed were collected (training phase), and a polynomial model was determined by fitting the mean value of ANA with respect to the walking speed using a least squares approach. This method was derived based on the fact that the force exerted by an object is directly proportional to the acceleration and the physical activity intensity (or walking speed) can be expressed as a function of acceleration. The overall mean squared error was 1.76 *km/h* based on the walking trials at speed ranging from 1 *km/h* to 13 *km/h*.

The implementation and the experimental results of these three studies demonstrated the feasibility of the abstraction model based method in estimating walking speed. As an inherent property of the abstraction model based method, the relationship between the walking speed and the inertial sensor measurements is modeled as black-box model with a set of parameters. Although the parameters do not have any explicit meaning, a map can be established through training. Although the off-line training could be time consuming, the walking speed estimation method itself is fast and highly simplified, which is suitable for real time implementation. Additionally, since no physical model is required in this method, a large variety of signals can potentially be used as inputs to the abstraction model, which implies that the sensor location and type are highly flexible (e.g., attached to back of the trunk and heel in [11] and to chest in [10]). Due to the fact that the abstraction model is an approximation of the actual physical system, the accuracy of the walking speed estimation depends on the completeness of training data set. The accuracy of the estimation is generally low and it is difficult to control the performance consistency across multiple subjects.

#### 3.4.2. Human Gait Model

Some researchers chose to estimate walking speed with stride length, based on some predefined human gait models. This class of methods was motivated by the fact that some aspects of the lower limb kinematics, other than stride length, can be determined from the measurements of the inertial sensor attached to the leg. With the assistance of a human gait model, the stride length can be indirectly estimated based on the measured lower limb kinematics.

Most earlier studies tried to employ a simplified gait model to avoid complicated sensor configuration and to reduce the computation complexity. In 1997, Miyazaki [20] proposed a stride length and walking speed estimation method using a gyroscope (uniaxial) attached to the thigh and a symmetric gait model. In this method, each leg was modeled as one single segment, and the two legs were assumed symmetrical; thus, at heel-strike, two legs and the distance between the feet formed a isosceles triangle. The angle between the leg segments was calculated by integrating the angular velocity measurement from the gyroscope attached to the thigh, and then the distance between two feet (step length, one half of stride length) was calculated using the properties of isosceles triangle. The overground walking experiment showed that this simple gait model method achieved a relative error of 15%. Similar to [20], Tong *et al.* [19] also modeled each leg as one segment and attached a gyroscope (uniaxial) on the shank. When calculating the stride length, Tong implicitly used a pendulum model in which the one-segment leg swung back and forth about the hip joint during walking. The stride length was simply calculated as the product of the leg segment inclination range (*rad*) and the leg segment length (*m*). Another method of using the single segment gait model was proposed by Tanaka *et al.* [25]. One additional accelerometer (biaxial) was attached to the shank along with a gyroscope (uniaxial). The algorithm used the same model as Miyazaki's method, but introduced the acceleration measurement to determine initial thigh angle just before walking initiation. In 2002, Aminian *et al.* [17] utilized a foot switch (to monitor temporal parameter) and two gyroscopes (uniaxial) to estimate walking speed. Discarding the simplified gait model reported in [19,20], Aminian *et al.* chose to solve the complete gait model with separate shank and thigh segments with the same assumption of symmetry between two legs. In this method, the rotation angles of the shank and the thigh were tracked by two gyroscopes, and each stride cycle was divided into stance phase and swing phase using the foot switch. The travel distance in each phase was solved geometrically using the rotation angles and the lengths of shank and thigh. Evaluated from both treadmill and overground walking experiments, the overall RMSE was 0.06 *m/s* (6.7%) for walking speed estimation and 0.07 *m* (7.2%) for stride length estimation. A different model of human gait was first introduced by Zijlstra *et al.* [12] in walking speed estimation. The proposed method, rather than attaching sensors on the lower limb, used the vertical displacement of the center of mass (CoM) to estimate walking speed. Since the CoM movements in the sagittal plane follow a circular trajectory about the foot during each single support phase, upon the determination of the CoM vertical displacement, the stride length can be derived geometrically. The experimental results showed that the maximum relative error is about 16%.

Using a predefined gait model along with inertial sensor measurements to estimate walking speed is beneficial in several aspects: (1) simple sensor setup, (2) ease of use. First, with the support of a gait model, the inertial sensors were usually used to provide only one or two kinematic parameters, e.g., the shank/thigh angle [17,19,20,25] and the vertical displacement of the CoM [12]. Since only one or two sensor measurements were processed in this method, less effort was required to deal with the inevitable sensor errors, *i.e.*, noise and bias. Second, unlike the abstraction model method, the gait model based method followed physical principles to construct the human gait model; thus, no subject-specific training phase was required before the actual walking speed estimation application. This method also has some shortcomings. The accuracy highly depends on the validity of the model, and the gait model directly affects the complexity of the algorithm. Comparing the performance of [17] and [20], the walking speed estimation error of the simplified gait model was about two times bigger than that of a more complete gait model. However, the improved accuracy required a much more complicated calculation procedure [17]. In addition, subject-specific anthropometric measurements, *i.e.*, lower limb segment length, must be taken in order to construct the human gait model.

#### 3.4.3. Direct Integration

In recent years, more and more studies started to use direct integration method to estimate walking speed. A generic direct integration algorithm includes the following steps: (1) define a starting and ending point of each stride cycle; (2) determine the orientation of the inertial sensor with respect to the global coordinate system; (3) project the acceleration measurement into the global coordinate system based on the instantaneous orientation of the inertial sensor and remove the acceleration due to gravity; and (4) integrate the acceleration in the global coordinate system from the starting point to obtain instantaneous sensor velocity and the associated stride length.

The direct integration methods have been developed separately for human gait analysis and personal navigation. Early studies using body-fixed inertial sensors mostly focused on human gait analysis. In the reviewed articles, the first direct integration walking speed estimation method was proposed by Sabatini *et al.* [26] in 2005, which used an IMU (biaxial accelerometer and biaxial of gyroscope) fixed on the instep of the foot. With a reasonable assumption that the foot (with the shoe) was rigid enough, the estimated sensor velocity could be viewed as the velocity of the foot. The foot flat (FF) event was defined as the starting point of each stride cycle and the angular velocity data was used to detect the FF event in the stance phase. One important procedure in this algorithm was the zero velocity update (ZUPT), which determined the initial sensor orientation and estimated the sensor measurement offsets during the period of the stride cycle. This method achieved an overall RMSE of 0.18 *km/h* based on the treadmill walking experiments at various speed ranging from 3 *km/h* to 6 *km/h*. Alvarez *et al.* [21] used a similar method to estimate the foot displacement over one stride cycle; however, Alvarez utilized one IMU (triaxial accelerometer and uniaxial gyroscope) on each foot and a data fusion algorithm to reduce the estimation error. Although the experimental results (relative error 10.1 ± 6.2%) showed limited improvement from the results of [26], Alvarez extended Sabatini's study and introduced a method to combine the information obtained from multiple sensors that could potentially increase the walking speed estimation accuracy. Attaching the sensor to the foot provided a lot of benefits, such as the possibility to implement ZUPT at foot flat, but the flexibility of the ankle joint brought concerns the influence of the abnormal gait on the inertial sensor data, such as out of plane motion [28]. To avoid such issue, Li *et al.* [27] attached an IMU (biaxial accelerometer and uniaxial gyroscope) to the lateral side of mid-shank and estimated the walking speed with a direct integration method. Different from attaching sensor on the foot, ZUPT technique could not be used for the sensor attached to the shank. Instead, they defined the start and end points of each stride cycle as when the shank was vertical (shank angle zero), and made use of the inverted pendulum model to assume that the initial sensor velocity was zero. This assumption is based on the fact that the CoM was at its highest point and the kinetic energy was all transformed into potential energy at the shank vertical event. In their study, a percentage RMSEs of 7% and 4% were obtained from the treadmill and the overground walking experiments, respectively. These methods [21,26,27] focused on walking speed estimation in the sagittal plane (or direction of progression) only, since most biomechanical studies used walking speed evaluated along a straight line, e.g., 10-meter walking test (10MWT) [29]. In 2010, Mariani *et al.* [18] attempted to use an IMU (triaxial accelerometer and triaxial gyroscope) attached to the back of the heel to estimate the stride length, stride velocity and turning angle in three-dimensional space. Quaternion representation of the sensor orientation in three-dimensional space was first obtained. The acceleration measurement was then projected to the global coordinate system based on the sensor orientation, and the acceleration due to gravity was removed from the projected sensor acceleration measurement. After the double integration of the projected sensor accelerations, the foot position in each stride was expressed in a 3-dimensional space. The stride length was determined as the distance between the positions of the foot at two adjacent foot flat events. The stride length and stride velocity estimation relative error were achieved as 1.3 ± 6.8% and 1.5 ± 5.8%, respectively.

In parallel to human gait analysis application, miniature inertial sensors have been used in personal navigation as a potential alternative to the GPS system. One attempt was conducted by Ojeda and Borenstein [22]. They developed a navigation system using an IMU (triaxial accelerometer and triaxial gyroscope) attached to the lateral side of the foot. They also used the quaternion representation of the sensor orientation to determine the instantaneous sensor orientation. The overall movement estimation was through a process called dead reckoning, with which the current position was determined by using a previously determined position. The travel distance estimation error was less than 2% as reported in [22]. In 2010, Martin *et al.* [14] used an IMU's (triaxial accelerometer and triaxial gyroscope) and two force sensors attached beneath the heel and the forefoot. The force sensors were used to detect the time instant of heel down (HD) that was defined as the starting point of the stride cycle. The overall stride length estimation error obtained from the 10 MWT was 34.1 ± 2.7 *mm*. Although the basic estimation procedure was the same, Huang *et al.* [24] used the direction cosines representation to track the orientation of the IMU (triaxial accelerometer and triaxial gyroscope) attached to the arch of the foot, and achieved a walking distance estimation error of about 2%. Moreover, concerning the effect of the sensor noise in the estimation accuracy, Bebek *et al.* employed a extended Kalman filter (EKF) to reduce the sensor noise and bias through the stance phase. A pressure sensor array was place between the heel of the shoe and the shoe insole, and an IMU (triaxial accelerometer and triaxial gyroscope) was attached to the lateral side of the foot. The pressure sensor array was used to detect the zero velocity period of the stride cycle, in which the ZUPT with EKF was applied. The relative error of the system was 0.40%, evaluated in the outdoor walking experiments with an average distance of 1,215 *m*.

The direct integration methods benefited from the increasing accuracy of the miniature IMUs and the sophisticated new algorithms. Compared to the abstraction model based method and the human gait model based method, this class of methods is easier to use without the troubles from the training process or subject-specific model building/calibration. Currently, the IMU is attached to the foot or the shank in order to take advantage of using the ground as a reference in the algorithm. As discussed in many studies, two important components of the direct integration method were the determination of the sensor orientation and the sensor error correction. Since the direct integration method solely used the IMU measurement in the estimation and very little external information was available on-the-fly, the sensor orientation determination heavily relied the angular velocity measurement or the angle representation such as Euler angle, quaternion and direction cosines. On the other hand, the sensor noise and bias must be reasonably corrected in the estimation process to ensure the accurate estimation results. One common sensor error correction technique was ZUPT. The sensor noise and bias were evaluated during the zero velocity period of the stride cycle, and then compensated from the calculation. With the direct integration method, three-dimensional motion monitoring was also made possible [14,18,22–24]. In contrast to human gait analysis application, personal navigation requires estimating 3D position based on only inertial sensor measurements and the accuracy of the estimation is critical because of a long duration operation. The reviewed articles indicate that the results from personal navigation applications tend to have better accuracies. Although most age-related or pathological gait research still considered walking speed during the straight line walking, the three-dimensional motion tracking capability will definitely extend the application of inertial sensors in human gait analysis.

## 4. Discussion

For the past two decades, a large amount of work has attempted to use inertial sensors in estimating walking speed. The existing methods can be categorized into three groups: abstraction model, human gait model and direction integration. Each method has its merits and limitations. There is a clear trend that more and more methods use the direct integration method with 2D or 3D IMUs. In the direct integration method, the double integration process amplifies measurement errors, leading to the requirement of inertial sensors with higher accuracy. Although the sensor performance has been ramped up dramatically, the inertial sensor measurement error is unavoidable, especially for miniature MEMS sensors. As one of the future research directions, development should focus on sensor error modeling and accommodation to further improve parameter estimation accuracy [30]. A method that combines human gait model and direct integration may be a potential candidate for this purpose. Since this systematic review is developed with a focus on algorithm development, performance evaluation or system validation articles were not included. As found from the reviewed studies, the experimental designs and the presentation of results are quite different between studies, which made comparison between methods difficult. A standard experimental benchmark or performance evaluation protocol should be developed to verify the performance of a new algorithm. Another area of algorithm development should focus on the unlevel ground walking and population with pathological gait. There has been a recent algorithm development for inclined walking [31]. Similarly, Using inertial sensors in estimating gait other than walking (e.g., running [32]) is also very interesting. As a limitation and the inherent nature of a systematic review, it is unavoidable to omit relevant literature due to the choice of keywords in the search. However, this review did cover the major methods in the area of inertial sensor based walking speed estimation methods and provide useful information for future algorithm development.

## Appendix

**Table 1.** Inertial Sensor Specifications in Reviewed Studies.

**Source**	**Year**	**Sensor Model**	**Number of Sensors**	**Type and Specification**				**Sampling Frequency** (*Hz*)	**Data Filtering** (*Hz*)
				**Accelerometer**		**Gyroscope**			
				**Measuring Axes**	**Measuring Range**	**Measuring Axes**	**Measuring Range**		
Aminian *et al.* [11]	1995	IC Sensors 3021	4	3				40	16
Miyazaki [20]	1997	ENC-05S, Murata	1			1	±150°*/s*		
Tong and Granat [19]	1999	ENC-05EA, Murata	1			1		50	0.3∼4
Aminian *et al.* [17]	2002	ENC-03J, Murata	3			1		200	
Zijlstra and Hof [12]	2003	Kistler	1	3	±2*g*			100	20
Tanaka *et al.* [25]	2004	acc: MC301, Wacoh gyro: ENC-03J, Murata	3			1		25	acc: 3 gyro: 0.3∼20
Sabatini *et al.* [26]	2005	acc: ADXL210E, Analog Device gyro: ENC-03J, Murata	1	2	±10*g*	2	±300°*/s*	200	acc: 17 gyro: 15
Alvarez *et al.* [21]	2007	MTx, Xsens	2	3	±2*g*	1		100	
Ojeda and Borenstein [22]	2007	SiIMU01, BAE	1	3	±50*g*	3	±1000°*/s*		
Song *et al.* [10]	2007	ADXL330	1	3	±3*g*			200	
Yeoh *et al.* [13]	2007	Crossbow	3	2	±2*g*			25	
Martin *et al.* [14]	2010	MTx, Xsens	2	3		3		50	15
Bebek *et al.* [23]	2010	InertiaCube3, InterSense	1	2	±6*g*	1			
Huang *et al.* [24]	2010		1	3		3			
Li *et al.* [27]	2010	acc: ADXL320 gyro: ADXRS300	1	2		1		1000	
Mariani *et al.* [18]	2010	S-Sense	1	3	±3*g*	3	roll, yaw: ±300°*/s* pitch: ±800°*/s*	200	17

* Empty entries indicate variables unspecified by the author.

**Table 2.** Sensor Type and Attachment Position. In total, nine different types of inertial sensors, including uniaxial, biaxial and triaxial accelerometer and gyroscope, were attached to 12 positions, including the chest, back of the trunk, thigh, shank and foot.

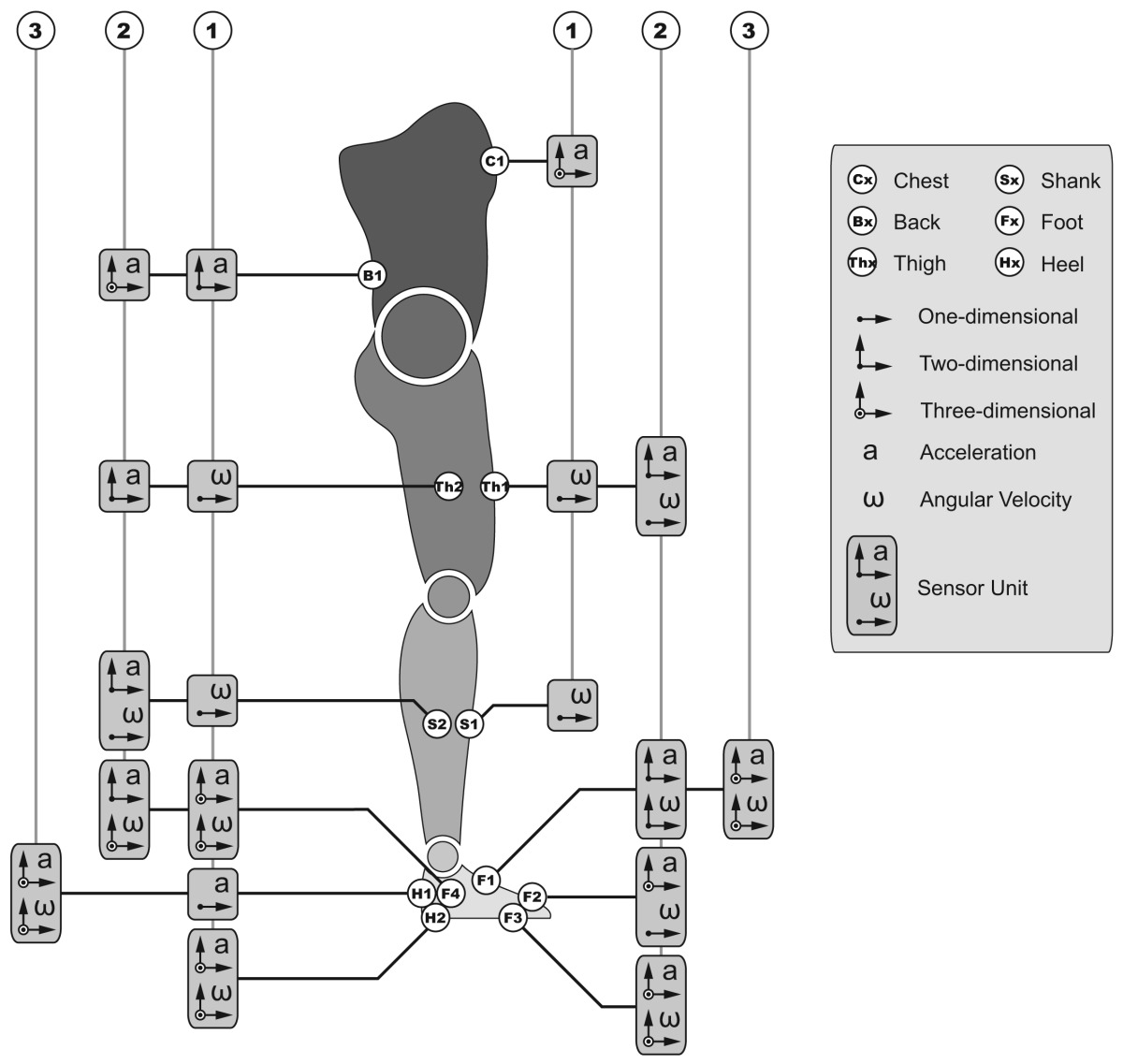

**Position**	**References**		

	1	2	3
C1	[10]		
B1	[13]	[11,12]	
Th1		[20]	
Th2	[17]	[13]	
S1	[19]		
S2	[17]	[27]	
F1		[26]	[24]
F2		[21]	
F3		[14]	
F4	[22]	[23]	
H1	[11]		[18]
H2	[14]		

**Table 3:** Experimental Design in Reviewed Studies.

**Source**	**Subject**	**Experimental Design**	
Aminian *et al.* [11]	5 healthy subjects	Treadmill walking at preferred speed.	

Miyazaki [20]	18 healthy subjects;	Healthy subjects:	Overground 25 m walking at preferred speed;
	7 patients with above knee prostheses;	Patients with above knee prostheses:	Overground 25 m walking at low, medium and high speed;
	10 hemiplegic subjects	Hemiplegic subjects:	Overground 15 m walking at low, medium and high speed.

Tong and Granat [19]	1 incomplete spinal cord injured subject;	Overground 4.5 m walking at preferred speed.	
	1 unimpaired subject		

Aminian *et al.* [17]	9 young subjects;	Young subjects:	Treadmill walking at preferred speed, under and over preferred speed;
	11 elderly subjects		Overground 30m walking at preferred speed
		Elderly subjects:	Overground 30m walking at preferred speed.

Zijlstra and Hof [12]	25 healthy subjects	Treadmill walking at 6 speed (0.5*m/s*, 0.75*m/s*, 1.0*m/s*, 1.25*m/s*, 1.5*m/s* and 1.75*m/s*);	
		Overground walking at preferred speed, slow and fast speed.	

Tanaka *et al.* [25]	10 healthy subjects	Overground walking at various speed.	

Sabatini *et al.* [26]	5 healthy subjects	Treadmill walking at combinations of 7 speed (3*km/s* to 6*km/s* in steps of 0.5*km/s*).	

Alvarez *et al.* [21]	1 healthy subject	Overground 10 m walking at preferred speed.	

Ojeda and Borenstein [22]	1 healthy subject	Overground walking at normal and brisk pace;	
		Overground walking along a square-shaped loop path on 1, 2 and 4 floors including stairs;	
		Overground 14-minute and 12-minute walking along city streets.	

Song *et al.* [10]	17 healthy subjects	Treadmill walking and running at various speed from 4.8*km/h* to 15.4*km/s*.	

Yeoh *et al.* [13]	5 healthy subjects	Treadmill walking and running at various speed from 1*km/h* to 13*km/s*.	

Martin *et al.* [14]	10 healthy subjects	Overground 10 m walking at preferred speed.	

Bebek *et al.* [23]	1 healthy subject	Overground half-hour walking along a loop.	

Huang *et al.* [24]	1 healthy subject	Overground walking along the square, rectangle, J-shaped paths and an athletic track.	

Li *et al.* [27]	8 healthy subjects	Treadmill walking at 6 speed (0.8*m/s*, 1.0*m/s*, 1.2*m/s*, 1.4*m/s*, 1.6*m/s* and 1.8*m/s*);	
		Overground 100 m walking at preferred speed.	

Mariani *et al.* [18]	10 young subjects; 10 elderly subjects	Overground 5 m “U-turn”, 3 m “8-turn” and 25 m 6-minute walking.	
